# Gas-Forming Hepatic Metastases Due to *Clostridium septicum* Infection: A Rare Case Documented with Sequential CT Imaging

**DOI:** 10.5334/jbsr.4079

**Published:** 2025-09-11

**Authors:** Mattia’s Loverde, Dan Truc Nguyen, Nasroola Damry

**Affiliations:** 1 Department of Radiology, CHU Brugmann, Brussels, Belgium; 2 Department of Oncology, CHU Brugmann, Brussels, Belgium

**Keywords:** Clostridium septicum, liver neoplasms/secondary, colonic neoplasms/complications, bacteremia

## Abstract

A rare case of hepatic gas-forming metastases secondary to *Clostridium septicum* infection is described. A 78-year-old woman was admitted with right lower quadrant abdominal pain and deterioration. Initial imaging revealed a right-sided colonic malignancy with hepatic metastases. Follow-up scans showed gas within metastases and minimal pneumoperitoneum, suggesting bowel perforation. However, blood cultures isolated *C. septicum*, supporting infected necrotic metastases as the source of free gas.

*Teaching point:* This case emphasizes the importance of distinguishing infectious intratumoral gas from perforation in oncological patients and provides one of the few reports with sequential imaging of this process.

## Introduction

*Clostridium septicum* is a spore-forming Gram-positive anaerobe strongly associated with gastrointestinal and hematologic malignancies [1–3]. While well described in soft tissues, hepatic gas-forming metastases remain rare and are seldom documented with sequential imaging [4, 5]. Other rare complications include aortitis and emphysematous hepatitis [6, 7]. Presented is a case with infected hepatic metastases, confirmed by blood cultures and repeated CT after suspicion of perforation. The transformation of hepatic metastases in the setting of *C. septicum* bacteremia is reported, with CT features of pre- and post-infectious stages.

## Case Report

A 78-year-old woman without an oncological history was referred to the emergency department on July 9, 2025, due to right lower quadrant abdominal pain and general deterioration. She reported fatigue, anorexia, and fever (39.6°C), without diabetes, corticosteroid use, or prior chemotherapy.

She was malnourished with sarcopenia and acute confusion. On clinical examination, there was tenderness and guarding in the right flank, without signs of acute abdomen. A firm, palpable mass was noted in the right iliac fossa.

Laboratory results showed leukocytosis (23,060/µL), high CRP (186 mg/L), thrombocytosis, anemia, and moderate renal dysfunction. Hepatic enzymes were slightly elevated (ALAT 49 U/L, ALP 226 U/L, GGT 200 U/L, LDH 322 U/L).

**Figure 1:**
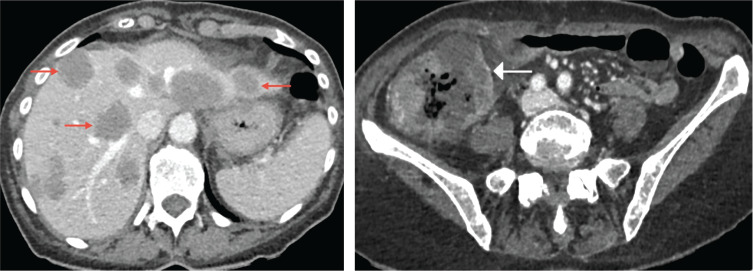
First contrast-enhanced CT scan (July 9, 2025) showing a thickened ascending colon (white arrow) and hypodense hepatic lesions without gas (red arrows).

CT showed an ascending colonic mass with cecal dilatation (7 cm), multiple hypodense hepatic metastases, and a small paracolic fluid collection, but no free air (Figure 1, first CT).

**Figure 2:**
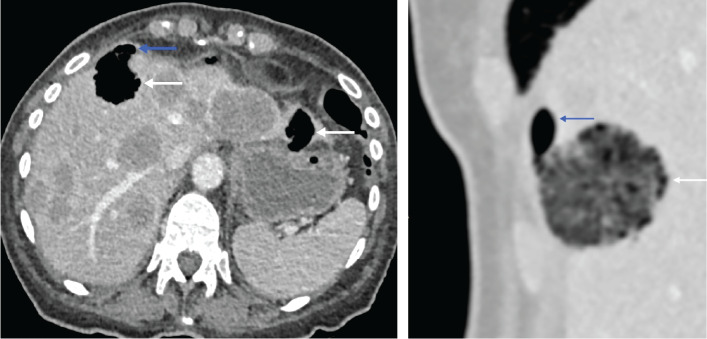
Follow-up CT scan (July 11, 2025) showing gas bubbles in several hepatic metastases (white arrows) and minimal pneumoperitoneum (blue arrows) in axial and sagittal planes.

Two days later, during the staging work-up, an unenhanced chest CT unexpectedly revealed the presence of air in several of the hepatic metastases as well as minimal pneumoperitoneum. This finding suggested bowel perforation, and an additional contrast-enhanced abdominal CT confirmed intratumoral gas within hepatic metastases and extension into the peritoneal cavity, indicating that the free gas originated from these lesions (Figure 2, follow-up CT).

Blood cultures (on July 10) showed *C. septicum*. Intravenous cefuroxime and metronidazole were initiated, later switched to oral therapy, with a favorable clinical evolution.

The final diagnosis was *C. septicum* bacteremia with infection of hepatic metastases and gas production, simulating a perforated colon. The patient refused any further diagnostic or therapeutic intervention. Consequently, no biopsy could be performed.

## Discussion

*C. septicum* infection is a known complication of colorectal cancer, particularly in right-sided tumors [1, 2, 8]. Its gas-generating properties can simulate bowel perforation or abscesses [3, 9]. Hepatic involvement remains rare, particularly in metastatic lesions, and very few reports include sequential imaging documentation [4, 5].

In this case, air in hepatic metastases and pneumoperitoneum initially suggested bowel perforation. However, blood cultures positive for *C. septicum* indicated bacterial translocation through a compromised mucosa due to subocclusion, with hematogenous seeding.

The right-sided tumor and subocclusion align with patterns described in the literature, associating *C. septicum* with colonic malignancies [2, 3]. Although histopathological confirmation was not obtained due to the patient’s refusal, the clinical, radiological, and microbiological findings strongly supported the presence of a right-sided colonic malignancy complicated by *C. septicum* bacteremia. The hematogenous spread is likely to be facilitated by tumor-induced tissue hypoxia and necrosis, which create an ideal anaerobic environment [1, 10].

The gas was confined to known liver metastases, suggesting microbial colonization and anaerobic metabolism in necrotic tumor tissue. This highlights the importance of considering infectious etiologies of intratumoral gas, especially when imaging findings are atypical.

This case documents, through sequential CT, the transformation of hepatic metastases into gas-containing lesions releasing gas into the peritoneum. This observation is rarely documented in the literature [2, 5, 8]. It supports infected metastases as the source of free air rather than perforation and objectivates the importance of early microbiological investigation in oncology patients with unusual metastatic imaging features. Early identification of *C. septicum* is crucial due to its high mortality. Broad-spectrum antibiotics with anaerobic coverage and surgical intervention when appropriate remain the basis of treatment [8].

## Conclusion

This case highlights a rare presentation of *C. septicum* bacteremia, in which gas formation in hepatic metastases produced pneumoperitoneum simulating bowel perforation. In patients with right-sided colonic tumors and anaerobic bacteremia, infectious transformation of metastases should be considered. Sequential CT imaging provides radiological insight into this transformation and emphasizes timely microbiological investigation of sepsis in an oncological setting.
